# Visualizing the effectiveness of face masks in obstructing respiratory jets

**DOI:** 10.1063/5.0016018

**Published:** 2020-06-01

**Authors:** Siddhartha Verma, Manhar Dhanak, John Frankenfield

**Affiliations:** Department of Ocean and Mechanical Engineering, Florida Atlantic University, Boca Raton, Florida 33431, USA

## Abstract

The use of face masks in public settings has been widely recommended by public health officials during the current COVID-19 pandemic. The masks help mitigate the risk of cross-infection via respiratory droplets; however, there are no specific guidelines on mask materials and designs that are most effective in minimizing droplet dispersal. While there have been prior studies on the performance of medical-grade masks, there are insufficient data on cloth-based coverings, which are being used by a vast majority of the general public. We use qualitative visualizations of emulated coughs and sneezes to examine how material- and design-choices impact the extent to which droplet-laden respiratory jets are blocked. Loosely folded face masks and bandana-style coverings provide minimal stopping-capability for the smallest aerosolized respiratory droplets. Well-fitted homemade masks with multiple layers of quilting fabric, and off-the-shelf cone style masks, proved to be the most effective in reducing droplet dispersal. These masks were able to curtail the speed and range of the respiratory jets significantly, albeit with some leakage through the mask material and from small gaps along the edges. Importantly, uncovered emulated coughs were able to travel notably farther than the currently recommended 6-ft distancing guideline. We outline the procedure for setting up simple visualization experiments using easily available materials, which may help healthcare professionals, medical researchers, and manufacturers in assessing the effectiveness of face masks and other personal protective equipment qualitatively.

Infectious respiratory illnesses can exact a heavy socio-economic toll on the most vulnerable members of our society, as has become evident from the current COVID-19 pandemic.^1,2^ The disease has overwhelmed healthcare infrastructure worldwide,^3^ and its high contagion rate and relatively long incubation period^4,5^ have made it difficult to trace and isolate infected individuals. Current estimates indicate that about 35% of infected individuals do not display overt symptoms^6^ and may contribute to the significant spread of the disease without their knowledge. In an effort to contain the unabated community spread of the disease, public health officials have recommended the implementation of various preventative measures, including social-distancing and the use of face masks in public settings.^7^

The rationale behind the recommendation for using masks or other face coverings is to reduce the risk of cross-infection via the transmission of respiratory droplets from infected to healthy individuals.^8,9^ The pathogen responsible for COVID-19 is found primarily in respiratory droplets that are expelled by infected individuals during coughing, sneezing, or even talking and breathing.^10–15^ Apart from COVID-19, respiratory droplets are also the primary means of transmission for various other viral and bacterial illnesses, such as the common cold, influenza, tuberculosis, SARS (Severe Acute Respiratory Syndrome), and MERS (Middle East Respiratory Syndrome), to name a few.^16–19^ These pathogens are enveloped within respiratory droplets, which may land on healthy individuals and result in direct transmission, or on inanimate objects, which can lead to infection when a healthy individual comes in contact with them.^10,18,20,21^ In another mode of transmission, the droplets or their evaporated contents may remain suspended in the air for long periods of time if they are sufficiently small. This can lead to airborne transmission^19,22^ when they are breathed in by another person, long after the infected individual may have left the area.

Several studies have investigated respiratory droplets produced by both healthy and infected individuals when performing various activities. The transport characteristics of these droplets can vary significantly depending on their diameter.^23–28^ The reported droplet diameters vary widely among studies available in the literature and usually lie within the range 1 *µ*m–500 *µ*m,^29^ with a mean diameter of ∼10 *µ*m.^30^ The larger droplets (diameter >100 *µ*m) are observed to follow ballistic trajectories under the effects of gravity and aerodynamic drag.^20,31^ Intermediate-sized droplets^20,31,32^ may get carried over considerable distances within a multiphase turbulent cloud.^33–35^ The smallest droplets and particles (diameter < 5 *µ*m–10 *µ*m) may remain suspended in the air indefinitely, until they are carried away by a light breeze or ventilation airflow.^20,32^

After being expelled into the ambient environment, the respiratory droplets experience varying degrees of evaporation depending on their size, ambient humidity, and temperature. The smallest droplets may undergo complete evaporation, leaving behind a dried-out spherical mass consisting of the particulate contents (e.g., pathogens), which are referred to as “droplet nuclei.”^36^ These desiccated nuclei, in combination with the smallest droplets, are potent transmission sources on account of two factors: (1) they can remain suspended in the air for hours after the infected individual has left the area, potentially infecting unsuspecting individuals who come into contact with them and (2) they can penetrate deep into the airways of individuals who breathe them in, which increases the likelihood of infection even for low pathogen loads. At present, the role of droplet nuclei in the transmission of COVID-19 is not known with certainty and the matter is the subject of ongoing studies.^37–39^ In addition to generating microscopic droplets, the action of sneezing can expel sheet-like layers of respiratory fluids,^40^ which may break apart into smaller droplets through a series of instabilities. The majority of the fluid contained within the sheet falls to the ground quickly within a short distance.

Regardless of their size, all droplets and nuclei expelled by infected individuals are potential carriers of pathogens. Various studies have investigated the effectiveness of medical-grade face masks and other personal protective equipment (PPE) in reducing the possibility of cross-infection via these droplets.^13,33,41–47^ Notably, such respiratory barriers do not prove to be completely effective against extremely fine aerosolized particles, droplets, and nuclei. The main issue tends to be air leakage, which can result in aerosolized pathogens being dispersed and suspended in the ambient environment for long periods of time after a coughing/sneezing event has occurred. A few studies have considered the filtration efficiency of homemade masks made with different types of fabric;^48–51^ however, there is no broad consensus regarding their effectiveness in minimizing disease transmission.^52,53^ Nonetheless, the evidence suggests that masks and other face coverings are effective in stopping larger droplets, which, although fewer in number compared to the smaller droplets and nuclei, constitute a large fraction of the total volume of the ejected respiratory fluid.

While detailed quantitative measurements are necessary for the comprehensive characterization of PPE, qualitative visualizations can be invaluable for rapid iteration in early design stages, as well as for demonstrating the proper use of such equipment. Thus, one of the aims of this Letter is to describe a simple setup for visualization experiments, which can be assembled using easily available materials. Such setups may be helpful to healthcare professionals, medical researchers, and industrial manufacturers, for assessing the effectiveness of face masks and other protective equipment qualitatively. Testing designs quickly and early on can prove to be crucial, especially in the current pandemic scenario where one of the central objectives is to reduce the severity of the anticipated resurgence of infections in the upcoming months.

The visualization setup used in the current study is shown in Fig. 1 and consists of a hollow manikin head which was padded on the inside to approximate the internal shape and volume of the nasal- and buccal-cavities in an adult. In case a more realistic representation is required, such a setup could include 3D-printed or silicone models of the internal airways. The manikin was mounted at a height of ∼5 ft and 8 in. to emulate respiratory jets expelled by an average human male. The circular opening representing the mouth is 0.75 in. in diameter. The pressure impulse that emulates a cough or a sneeze may be delivered via a manual pump, as shown in Fig. 1, or via other sources such as an air compressor or a pressurized air canister. The air capacity of the pump is 500 ml, which is comparable to the lower end of the total volume expelled during a cough.^54^ We note that the setup here emulates a simplified representation of an actual cough, which is an extremely complex and dynamic problem.^55^ We use a recreational fog/smoke machine to generate tracer particles for visualizing the expelled respiratory jets, using a liquid mixture of distilled water (4 parts) and glycerin (1 part). Both the pressure- and smoke-sources were connected to the manikin using clear vinyl tubing and NPT fittings wherever necessary.

**FIG. 1.**
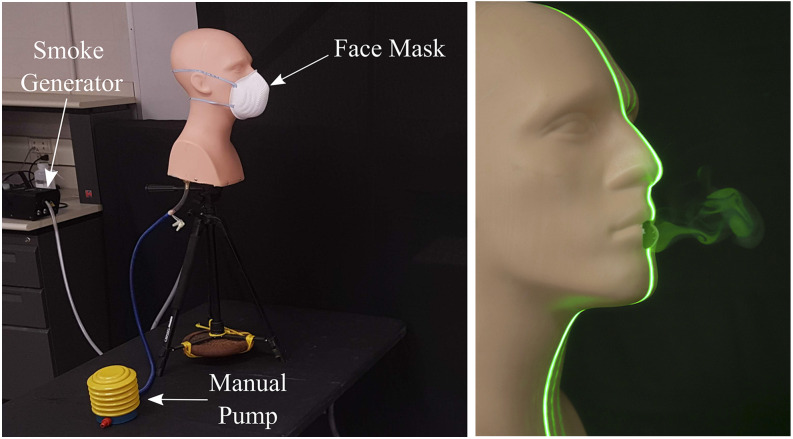
Left—experimental setup for qualitative visualization of emulated coughs and sneezes. Right—a laser sheet illuminates a puff emerging from the mouth.

The resulting “fog” or “smoke” is visible in the right panel of Fig. 1 and is composed of microscopic droplets of the vaporized liquid mixture. These are comparable in size to the smallest droplets expelled in a cough jet (∼1 *µ*m–10 *µ*m). We estimate that the fog droplets are less than 10 *µ*m in diameter, based on Stokes’ law and our observation that they could remain suspended for up to 3 min in completely still air with no perceptible settling. The laser source used to generate the visualization sheet is an off-the-shelf 5 mW green laser pointer with 532 nm wavelength. A plane vertical sheet is created by passing the laser beam through a thin cylindrical rod (diameter 5 mm) made of borosilicate glass.

We first present visualization results from an emulation of an uncovered heavy cough. The spatial and temporal evolution of the resulting jet is shown in Fig. 2. The aerosolized microscopic droplets visible in the laser sheet act as tracer particles, revealing a two-dimensional cross section of the conical turbulent jet. These tracers depict the fate of the smallest ejected droplets and any resulting nuclei that may form. We observed high variability in droplet dispersal patterns from one experimental run to another, which was caused by otherwise imperceptible changes in the ambient airflow. This highlights the importance of designing ventilation systems that specifically aim to minimize the possibility of cross-infection in a confined setting.^23,56–58^

**FIG. 2.**
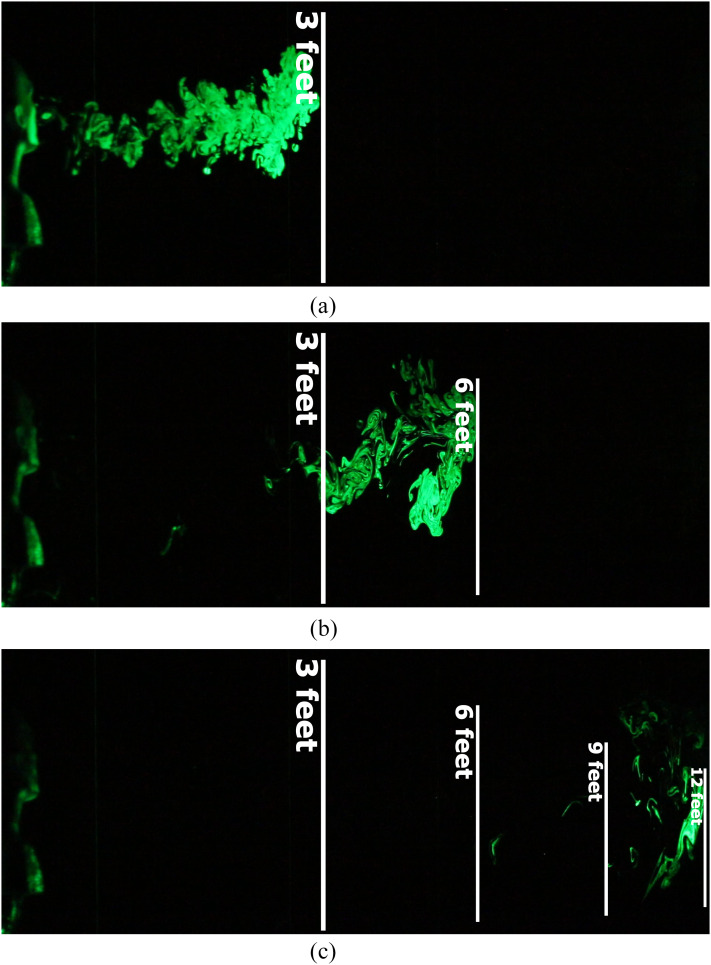
An emulated heavy cough jet travels up to 12 ft in ∼50 s, which is twice the CDC’s recommended distancing guideline of 6 ft.^7^ Images taken at (a) 2.3 s, (b) 11 s, and (c) 53 s after the initiation of the emulated cough.

Despite high variability, we consistently observed jets that traveled farther than the 6-ft minimum distance proposed by the U.S. Centers for Disease Control and Prevention (CDC’s).^7^ In the images shown in Fig. 2, the ejected tracers were observed to travel up to 12 ft within ∼50 s. Moreover, the tracer droplets remained suspended midair for up to 3 min in the quiescent environment. These observations, in combination with other recent studies,^35,59^ suggest that current social-distancing guidelines may need to be updated to account for the aerosol-based transmission of pathogens. We note that although the unobstructed turbulent jets were observed to travel up to 12 ft, a large majority of the ejected droplets will fall to the ground by this point. Importantly, both the number and concentration of the droplets will decrease with increasing distance,^59^ which is the fundamental rationale behind social- distancing.

We now discuss dispersal patterns observed when the mouth opening was blocked using three different types of face masks. For these results, we focus on masks that are readily accessible to the general public, which do not draw away from the supply of medical-grade masks and respirators for healthcare workers. Figure 3 shows the impact of using a folded cotton handkerchief mask on the expelled respiratory jet. The folded mask was constructed by following the instructions recommended by the U.S. Surgeon General.^60^ It is evident that while the forward motion of the jet is impeded significantly, there is notable leakage of tracer droplets through the mask material. We also observe a small amount of tracers escaping from the top edge of the mask, where gaps exist between the nose and the cloth material. These droplets remained suspended in the air until they were dispersed by ambient disturbances. In addition to the folded handkerchief mask discussed here, we tested a single-layer bandana-style covering (not shown) which proved to be substantially less effective in stopping the jet and the tracer droplets.

**FIG. 3.**
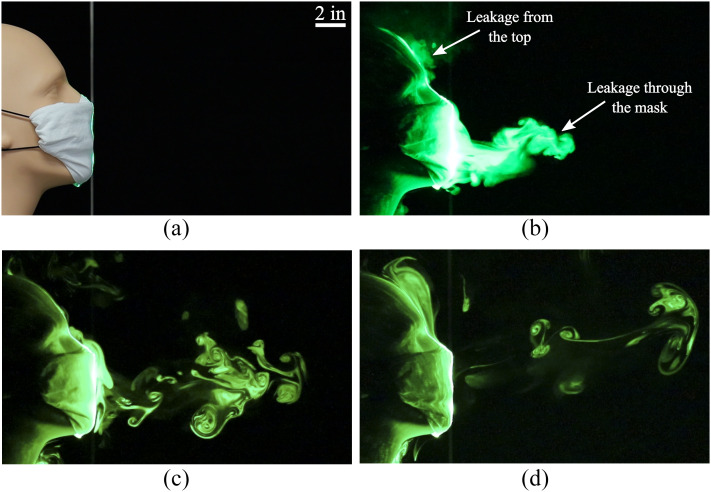
(a) A face mask constructed using a folded handkerchief. Images taken at (b) 0.5 s, (c) 2.27 s, and (d) 5.55 s after the initiation of the emulated cough.

We now examine a homemade mask that was stitched using two-layers of cotton quilting fabric consisting of 70 threads/in. The mask’s impact on droplet dispersal is shown in Fig. 4. We observe that the mask is able to arrest the forward motion of the tracer droplets almost completely. There is minimal forward leakage through the material, and most of the tracer-escape happens from the gap between the nose and the mask along the top edge. The forward distance covered by the leaked jet is less than 3 in. in this case. The final mask design that we tested was a non-sterile cone-style mask that is available in most pharmacies. The corresponding droplet-dispersal visualizations are shown in Fig. 5, which indicate that the flow is impeded significantly compared to Figs. 2 and 3. However, there is noticeable leakage from gaps along the top edge. The forward distance covered by the leaked jet is ∼6 in. from the mouth opening, which is farther than the distance for the stitched mask in Fig. 4.

**FIG. 4.**
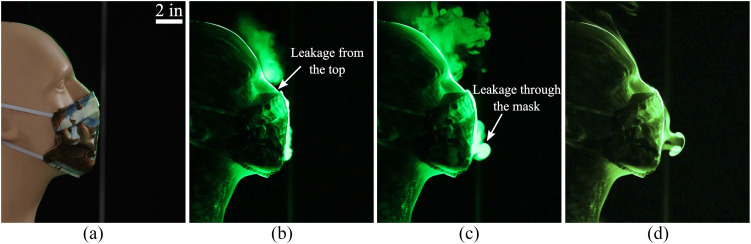
(a) A homemade face mask stitched using two-layers of cotton quilting fabric. Images taken at (b) 0.2 s, (c) 0.47 s, and (d) 1.68 s after the initiation of the emulated cough.

**FIG. 5.**
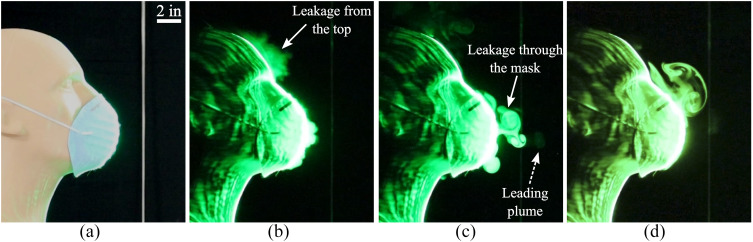
(a) An off-the-shelf cone style mask. (b) 0.2 s after initiation of the emulated cough. (c) 0.97 s after initiation of the emulated cough. The leading plume, which has dissipated considerably, is faintly visible. (d) 3.7 s after initiation of the emulated cough.

A summary of the various scenarios examined in this study is provided in Table I, along with details about the mask material and the average distances traveled by the respiratory jets. We observe that a single-layer bandana-style covering can reduce the range of the expelled jet to some extent, compared to an uncovered cough. Importantly, both the material and construction techniques have a notable impact on the masks’ stopping-capability. The stitched mask made of quilting cotton was observed to be the most effective, followed by the commercial mask, the folded handkerchief, and, finally, the bandana. Importantly, our observations suggest that a higher thread count by itself is not sufficient to guarantee better stopping-capability; the bandana covering, which has the highest thread count among all the cloth masks tested, turned out to be the least effective.

**TABLE I.** A summary of the different types of masks tested, the materials they are made of, and their effectiveness in impeding droplet-dispersal. The last column indicates the distance traveled by the jet beyond which its forward progression stops. The average distances have been computed over multiple runs, and the symbol “∼” is used to indicate the presence of high variability in the first two scenarios listed.

Mask type	Material	Threads/in.	Average jet distance
Uncovered	…	…	∼8 ft
Bandana	Elastic T-shirt material	85	∼3 ft 7 in.
Folded handkerchief	Cotton	55	1 ft 3 in.
Stitched mask	Quilting cotton	70	2.5 in.
Commercial mask^a^	Unknown	Randomly assorted fibres	8 in.

^a^ CVS Cone Face Mask.

We note that it is likely that healthcare professionals trained properly in the use of high-quality fitted masks will not experience leakage to the extent that we have observed in this study. However, leakage remains a likely issue for members of the general public who often rely on loose-fitting homemade masks. Additionally, the masks may get saturated after prolonged use, which might also influence their filtration capability. We reiterate that although the non-medical masks tested in this study experienced varying degrees of flow leakage, they are likely to be effective in stopping larger respiratory droplets.

In addition to providing an initial indication of the effectiveness of protective equipment, the visuals used in this study can help convey to the general public the rationale behind social-distancing guidelines and recommendations for using face masks. Promoting widespread awareness of effective preventative measures is crucial, given the high likelihood of a resurgence of COVID-19 infections in the fall and winter.

## DATA AVAILABILITY

The data that support the findings of this study are available within this article.
